# MiR-18a affects hypoxia induced glucose metabolism transition in HT22 hippocampal neuronal cell line through the *Hif1a* gene

**DOI:** 10.1186/s12883-024-03717-w

**Published:** 2024-06-15

**Authors:** Chuncheng Liu, Gehui Liu, Xinyang Zuo, Donghui Qu, Yefeng Sun, Linan Liu, Xiujuan Zhao, Jun Li, Lu Cai

**Affiliations:** 1 School of Life Science and Technology, Inner Mongolia University of Science and Technology, Baotou, 014010 China; 2 Inner Mongolia Key Laboratory of Functional Genome Bioinformatics, Baotou, 014010 China

**Keywords:** Glucose metabolism, *Hif1a*, Hypoxia, miR-18a, Neuronal

## Abstract

Hypoxia can cause a variety of diseases, including ischemic stroke and neurodegenerative diseases. Within a certain range of partial pressure of oxygen, cells can respond to changes in oxygen. Changes in oxygen concentration beyond a threshold will cause damage or even necrosis of tissues and organs, especially for the central nervous system. Therefore, it is very important to find appropriate measures to alleviate damage. MiRNAs can participate in the regulation of hypoxic responses in various types of cells. MiRNAs are involved in regulating hypoxic responses in many types of tissues by activating the hypoxia-inducible factor (HIF) to affect angiogenesis, glycolysis and other biological processes. By analyzing differentially expressed miRNAs in hypoxia and hypoxia-related studies, as well as the HT22 neuronal cell line under hypoxic stress, we found that the expression of miR-18a was changed in these models. MiR-18a could regulate glucose metabolism in HT22 cells under hypoxic stress by directly regulating the 3’UTR of the *Hif1a* gene. As a small molecule, miRNAs are easy to be designed into small nucleic acid drugs, so this study can provide a theoretical basis for the research and treatment of nervous system diseases caused by hypoxia.

**Graphical Abstract:**

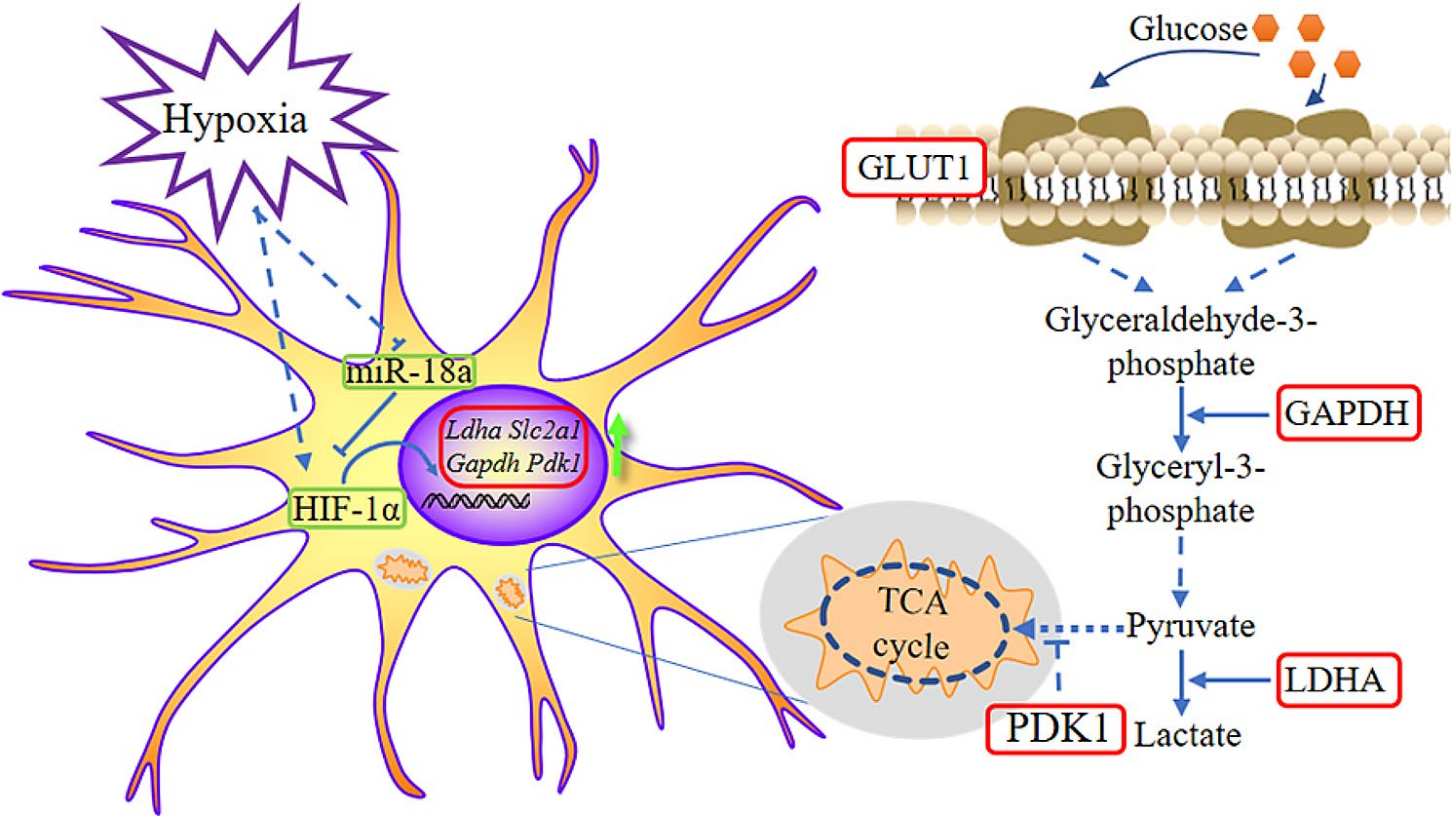

## Introduction

Hypoxia usually refers to the pathological phenomenon in which cells do not receive adequate oxygen supply due to multiple factors, or the oxygen in cells is insufficient to support metabolic functions. Hypoxia can cause a variety of disorders, including ischemic stroke, neurodegenerative diseases, tumor metastasis, cardiovascular disease, chronic kidney disease, metabolic diseases, preeclampsia, etc [1–3]. At the same time, physiological hypoxia is engaged in multiple normal developmental processes including stem cells stemness maintenance [4], embryonic development [5] and vascularization [6]. More than 20% of the body’s metabolism is used up by the central nervous system, while 75–80% of the energy used by the central nervous system is consumed by neurons. Consequently, the central nervous system is the tissue most vulnerable to hypoxia among other tissues [7].

Hypoxia induced alterations in glucose metabolism and mitochondrial function can cause neuronal dysfunction, structural damage, and neuronal death. In the central nervous system, the hippocampus is very sensitive to hypoxia, so hypoxia-mediated apoptosis and necrosis of hippocampus are often the main reasons for nerve function damage [1].

Hypoxic inducible factors (HIFs), as hypoxic sensing molecules at cellular and molecular levels, are the most critical regulatory proteins in hypoxic signaling pathways. HIFs include HIF-1, HIF-2 and HIF-3 subtypes. Each of HIFs is a heterodimeric protein composed of α and β subunits. Under normal oxygen conditions, HIF-1α is synthesized in the cytoplasm and recognized by ubiquitin ligase von Hippel–Lindau (VHL) tumor suppressor protein due to hydroxylation, and finally enters the proteasome degradation pathway. When the cell is in a hypoxic environment, HIF-1α can enter the nucleus and form heterodimer with HIF-1β, thus activating the transcription of downstream target genes [8]. HIF-1 controls the expression of more than 700 different target genes, and the major target genes are related to angiogenesis and energy metabolism. Of particular concern, HIF-1 regulates the expression of erythropoietin (EPO) and vascular endothelial growth factor (VEGF), as well as genes that regulate glucose transport or glucose metabolism, such as *SLC2A1*, *GAPDH*, *PDK1*, and *LDHA* [1, 9].

MiRNAs play an important regulatory role in the hypoxic response process caused by various factors such as tumor development, tissue damage repair, altitude sickness, and cardiovascular disease [10]. Among them, the expression of miR-18a was down-regulated in choroidal endothelial cells treated with hypoxia [11], breast cancer cells with lung metastases [12], and colorectal tumor cells under cobalt chloride stress [13]. At the same time, miR-18a is also engaged in regulating glucose metabolism and blood glucose [14, 15]. Further research is required to fully understand the function of miR-18 in neuronal response to hypoxia.

Our results in HT22 hippocampal neuronal cell line showed that hypoxic stress induced down-regulation of miR-18a, and the change of miR-18a had an impact on the expression of genes involved in glycolysis. In this process, the regulation of metabolism by miR-18a was related to its ability to inhibit the *Hif1a* gene. Bioinformatics analysis showed that the *Hif1a* gene is the direct target gene of miR-18a, and miR-18a could affect the expression of the *Hif1a* gene through the complementary sequence in the 3’UTR. In conclusion, we investigate the role of miR-18a in the regulation of the *Hif1a* gene and glucose metabolism in hypoxic stress HT22 cells. This study could provide a theoretical basis for the investigation and care of hypoxia-induced neuronal damage.

## Materials and methods

### Cell culture and treatments

HT22 is a murine hippocampal neuronal cell line. HT22 can be used as a model for studies relevant to hippocampal neurons in vitro [16]. HT22 cells were purchased from National Science & Technology Infrastructure—National BioMedical Cell-Line Resource of China. HT22 cells and HEK293T cells were cultured in DMEM medium containing 1% penicillin and streptomycin and 10% FBS in an incubator containing 5% CO_2_ at 37℃.

Under hypoxic stress, HT22 cells were cultured at 37℃ in an incubator containing 5% CO_2_ and 1% O_2_ for 12–48 h.

Cobalt chloride treatment was performed by culturing HT22 cells using medium containing 200 μm CoCl_2_ for 18 h.

### Transfections

Cells were transfected with liposome (Beyotime, China). HT22 cells were seeded in 6-well plates and transfected when the confluence of the cells was 50-60%. In each well, the transfection amount of miR-18a mimics or siRNA was 240 pmol. After 6 h of transfection, the medium was replaced with normal medium (for cobalt chloride treatment, the medium was replaced with medium containing cobalt chloride).

HEK293T cells were seeded in 24-well plates and transfected when the confluence of the cells was 60-70%. In each well, the transfection amount of miR-18a mimics and vector was 40 pmol and 500 ng. The relative luciferase activity was detected 24 h after transfection.

### Lactic acid determination

When the confluence of HT22 cells reached 70%, hypoxic stress was initiated. After 12 h and 24 h hypoxia, the lactic acid content of the cell culture medium was determined using the Lactic acid assay kit (Nanjing Jiancheng Bioengineering Institute, China). Lactic acid was examined according to the instruction manual, and the absorbance at 530 nm wavelength was finally measured by a microplate reader.

### Quantitative real-time polymerase chain reaction (qPCR)

Total RNA was extracted using TRIzol™ Reagent (Thermo Fisher Scientific, MA, USA) and Total RNApure Reagent (Beijing Zoman Biotechnology Co., Ltd., China). The primers and methods used for reverse transcription of miR-18a were as previously described [17].

The mRNA was reverse transcribed into cDNA by oligo(dT) and M-MLV Reverse Transcriptase (Promega, WI, USA). The expressions of miR-18a, *Hif1a*, *Ldha*, *Gapdh*, *Pdk1*, and *Slc2a1* (The protein corresponding to the *Slc2a1* gene is Glut1) were detected by quantitative real-time polymerase chain reaction (qPCR) using the SYBR Green PCR mix kit. The *Actb* gene was used as the internal reference gene (The protein corresponding to the *Actb* gene is β-actin), and the differential expression level was calculated by 2^−△△CT^.

The primers used are as follows:

*Ldha*, 5’-GCCACTGATTTTCCAAGCCA-3’ and 5’-GTCCAGCGAAACGTGAACAT-3’.

*Gapdh*, 5’-GGCTGCCCAGAACATCAT-3’ and 5’-CGGACACATTGGGGGTAG-3’.

*Pdk1*, 5’-GCTGAGAAGAATGGTGTGAAGATTA-3’ and.

5’-ATCCAGCCAGGTATGCCAGA − 3’

*Slc2a1*, 5’-TTGTTGTAGAGCGAGCTGGA-3’ and 5’-ATGGCCACGATGCTCAGATA-3’.

*Hif1a*, 5’-GCGGCGAGAACGAGAAGA-3’ and 5’-GTGGGGAAGTGGCAACTGAT-3’.

*Actb*, 5’- ACCCACACTGTGCCCATCTA − 3’ and 5’- CACGCTCGGTCAGGATCTTC − 3’.

### Western blot

HT22 cells were lysed using RIPA lysate containing phenylmethylsulfonyl fluoride. The specific process for protein detection was as described previously [18]. The following antibodies were used: anti-HIF1 alpha (1:1000) (GTX127309, GeneTex, TX), anti-GAPDH (1:800) (AF1186, Beyotime, China), anti-PDK1 (1:800) (AF7707, Beyotime, China), anti-LDHA (1:800) (AF0216, Beyotime, China), anti-β-actin (1:1500) (WH216231, ABclonal Technology Co.,Ltd., China).

### Vector construction and luciferase activity assays

The construction of vectors and the detection method of luciferase activity are as previously described [19].The main processes were as follows: The 3’UTR of the mouse *Hif1a* gene was amplified by PCR using the cDNA as template. The fragment was then cloned into the multiple cloning site of the psiCHECK^TM^-2 vector (Promega (Beijing) Biotech Co., Beijing, China) (named: psi-3’UTR-Hif1a). The predicted binding site of miR-18a on the 3’UTR of the *Hif1a* gene was mutated by fusion PCR. This fragment was also cloned into the multiple cloning site of the psiCHECK^TM^-2 vector (named: psi-3’UTR-mut-Hif1a). The regulatory effects of miR-18a on the corresponding sequences were finally determined by contrasting the relative activities of Renilla luciferase with Firefly luciferase.

### Data sources

Fei Han et al. examined the expression changes of miRNAs by microarray after choroidal endothelial cells were cultured under normoxic or hypoxic conditions and analyzed the differentially expressed miRNAs [11].

Raisa Krutilina et al. used microarray to analyze the differentially expressed miRNAs between breast cancer cells in situ and breast cancer cells with spontaneous lung metastasis [12].

Stepan Nersisyan et al. simulated the effect of the hypoxic microenvironment on tumor cells by stressing human colorectal cancer cell line with cobalt chloride. The expression of miRNA was detected using small RNA sequencing, and the differentially expressed miRNAs are provided in this manuscript [13].

Sukjun Kim et al. overexpressed a variety of miRNAs (including miR-18a) in HepG2 cell line respectively, and detected gene expression changes through RNA sequencing [20]. Based on the transcriptome data, differentially expressed genes caused by overexpression of miR-18a were screened by *p* adjust < 0.05 and fold change > 2.

### Statistical analysis

The predicted target genes of miR-18a were analyzed using miRWalk (Mouse, miRWalk version 3, University of Heidelberg, Heidelberg, Germany) and TargetScan7.2 (Mouse, version 7.2, Whitehead Institute for Biomedical Research, Boston, MA, USA).

Using Jvenn [21] to obtain the intersection between the predicted target genes of miR-18a and differentially expressed genes caused by miR-18a overexpression. The genes in this intersection were clustered and visualized by the clusterprofiler package in the R language.

The phylogenetic trees of the 3’UTR sequences of the *Hif1a* gene for human, chimpanzee, rhesus monkey, bovine, dog, rat, mouse, opossum, and chicken were constructed by MEGA 11 (Neighbor joining). The corresponding sequences were downloaded from the GenBank database. Sequences complementary to the miR-18a seed sequences and the flanking sequences in the 3’UTR of the *Hif1a* gene from multiple species were analyzed and visualized by weblogo (version 3, University of California, Berkeley, CA, USA).

Statistical analysis was performed using SPSS 16.0. The experimental results were presented as mean ± standard error (mean ± SEM), and the statistical significance was calculated by Student’s t-test. *p* < 0.05 was considered statistically significant. * indicates a significant difference (*p* < 0.05), ** indicates an extremely significant difference (*p* < 0.01).

## Results

### MiR-18a was involved in the regulation of glucose metabolism in HT22 cells under hypoxic stress

**Fig. 1:**
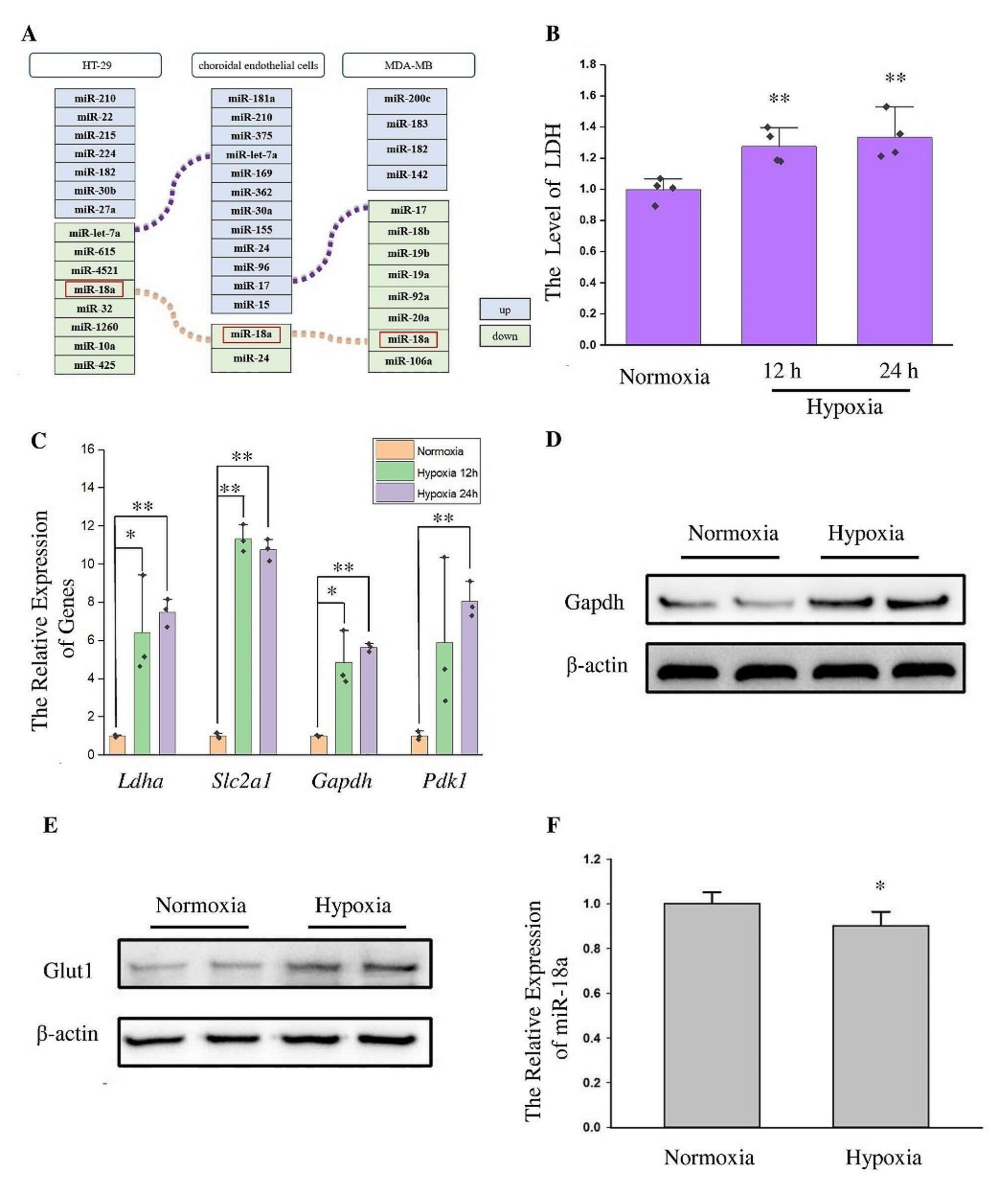
Hypoxic stress affected glucose metabolism and the expression of miR-18a in HT22 cells. (**A**) Analysis of differentially expressed miRNAs in hypoxia and hypoxia related studies. (**B**) The effect of hypoxic stress on lactic acid production was detected. *n* = 4, **: *p* < 0.01. (**C**) qPCR was used to detect the expression of *Ldha*, *Slc2a1*, *Gapdh* and *Pdk1* genes in HT22 cells after hypoxia stress. The *Actb* gene was used as the internal reference gene (the protein corresponding to the *Actb* gene is β-actin). *n* = 3, *: *p* < 0.05, **: *p* < 0.01. (**D**-**E**) Western blot was used to detect the expression of Gapdh and Glut1 after 24 h of hypoxic stress. β-actin was used as an internal reference protein. (**F**) The expression of miR-18a in HT22 cells after hypoxic stress was detected by qPCR. U6 gene was used as the internal reference gene. *n* = 3, *: *p* < 0.05

In order to study miRNAs that play a key regulatory role in the hypoxic response, we first analyzed the differentially expressed miRNAs from published hypoxic stress-related studies (Fig. 1A). These studies included the differentially expressed miRNAs induced by hypoxic stress in choroidal endothelial cells. Cobalt chloride treatment can simulate the hypoxic microenvironment, we also analyzed the differentially expressed miRNAs induced by cobalt chloride stress. Hypoxia is a key factor causing tumor metastasis [2], so we analyzed the differentially expressed miRNAs between breast cancer cells in situ and breast cancer cells that spontaneously metastasized to the lungs. In the three studies above, only the expression level of miR-18a changed in the all models.

To investigate the function of miR-18a, we firstly constructed a cell model of hypoxic stress in vitro using the HT22 hippocampal neuronal cell line. Lactic acid production was significantly increased in HT22 cells exposed to hypoxic stress for 12 and 24 h (Fig. 1B). The increase in lactic acid production may be due to the enhancement of glucose anaerobic oxidation. So we examined the expression of genes involved in glucose anaerobic oxidation by qPCR, including Glucose Transporter Type 1 (*Slc2a1*), Lactate Dehydrogenase (*Ldha*), Pyruvate Dehydrogenase Kinase, Isoenzyme 1 (*Pdk1*), Glyceraldehyde-3-Phosphate Dehydrogenase (*Gapdh*). The results showed that the mRNA levels of the above genes increased significantly after 12 h and 24 h hypoxic stress (Fig. 1C). The detection results at the protein level also proved that the expression levels of the anaerobic oxidation related protein Glut1 and Gapdh were increased after hypoxic stress (Fig. 1D, E).

After determining the response of neuronal cells to hypoxic stress, we examined the expression of miR-18a after hypoxic stress (Fig. 1F), and the findings indicated that miR-18a was down regulated after hypoxic treatment.

**Fig. 2:**
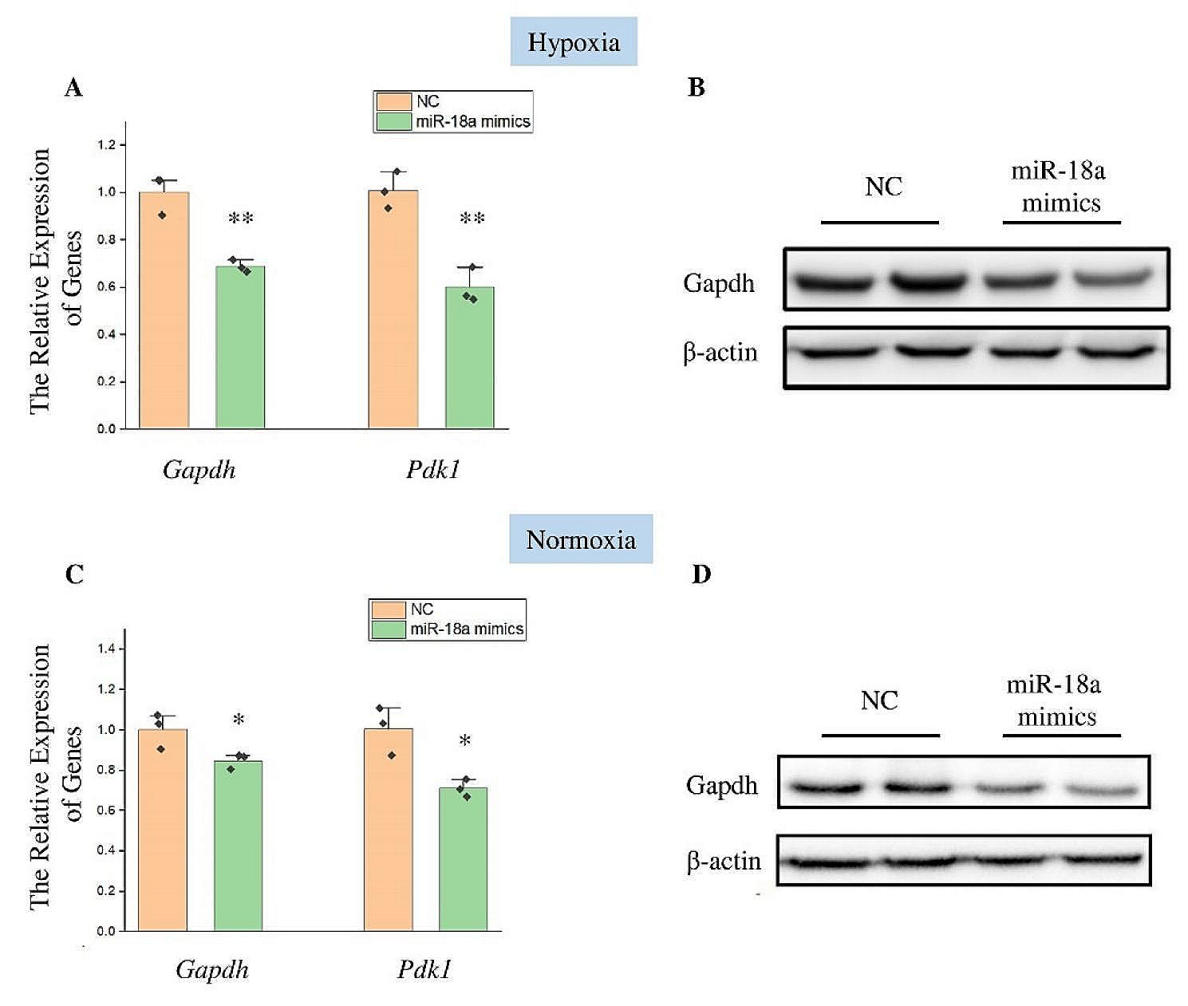
MiR-18a regulated the expression of genes related to glycolysis. (**A**) The expression of the *Gapdh* gene and *Pdk1* gene was detected by qPCR after transfection of miR-18a mimics and negative control (NC) for 24 h and hypoxic stress for 12 h. The *Actb* gene was used as the internal reference gene. *n* = 3, **: *p* < 0.01. (**B**) Western blot was used to detect the expression of Gapdh after transfection of miR-18a mimics and NC for 24 h and hypoxic stress for 12 h. β-actin was used as an internal reference protein. (**C**) The expression of the *Gapdh* gene and *Pdk1* gene was detected by qPCR after transfection of miR-18a mimics and NC for 36 h. The *Actb* gene was used as the internal reference gene. *n* = 3, *: *p* < 0.05. (**D**) Western blot was used to detect the expression of Gapdh after transfection of miR-18a mimics and negative control for 36 h. β-actin was used as an internal reference protein

Then, we investigated the function of miR-18a. HT22 cells were subjected to hypoxic stress after transfection with miR-18a mimics or negative control for 24 h. The *Gapdh* gene and *Pdk1* gene were detected after 12 h of hypoxic exposure (Fig. 2A). The qPCR results demonstrated that the overexpression of miR-18a could inhibit the *Gapdh* gene and *Pdk1* gene under hypoxic conditions. Gapdh was also detected at protein level, and the results were consistent with the findings at mRNA level (Fig. 2B). We also examined the effect of miR-18a on glycolysis under non-hypoxic stress. The expression of the *Gapdh* gene and *Pdk1* gene were examined after HT22 cells were transfected with miR-18a mimics or negative control (Fig. 2C). The results demonstrated that, in non-hypoxic conditions, miR-18a suppressed the expression of the *Gapdh* gene and *Pdk1* gene. Western blot results also indicated that miR-18a inhibited Gapdh without hypoxic stress (Fig. 2D).

### MiR-18a directly regulated the expression of the Hif1a gene

**Fig. 3:**
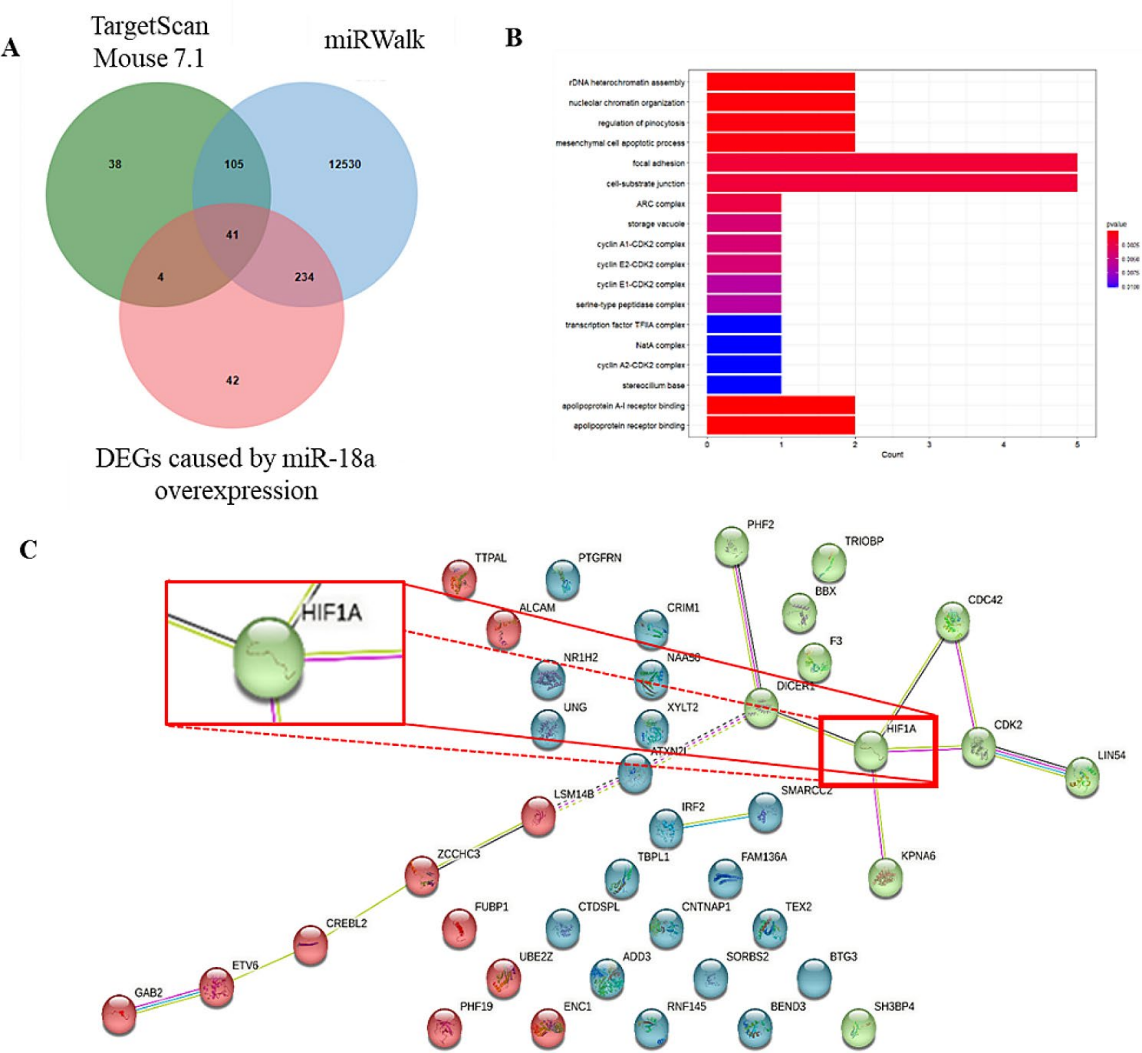
Prediction of miR-18a target genes. (**A**) The Venn diagram showed the intersection of miR-18a target genes predicted using TargetScanMouse 7.1, miR-18a target genes predicted using miRWalk, and differentially expressed genes caused by miR-18a overexpression. (**B**) Top 20 significantly enriched GO functional categories. (**C**) Analysis of protein-protein interactions

The biological functions of miRNAs are mainly achieved by regulation of the 3’UTR of target genes. TargetScanMouse 7.1 and miRWalk were used to identify the target genes that miR-18a may inhibit in order to ascertain the regulatory mechanism of miR-18a. Based on the published results [13], the differentially expressed genes induced by miR-18a overexpression were analyzed. The intersection of the differentially expressed genes and the predicted target genes was obtained by Venn diagram (Fig. 3A). There were 41 genes in the intersection.

In order to identify the key genes regulated by miR-18a, GO enrichment analysis of these 41 genes was performed (Fig. 3B). However, there were no metabolism related biological processes in the enrichment analysis results. Therefore, protein-protein interaction (PPI) was used to find the key genes among these 41 genes (Fig. 3C). The PPI result demonstrated the importance of Hif-1α. At the same time, several studies have reported that Hif-1α is closely related to metabolism.

**Fig. 4:**
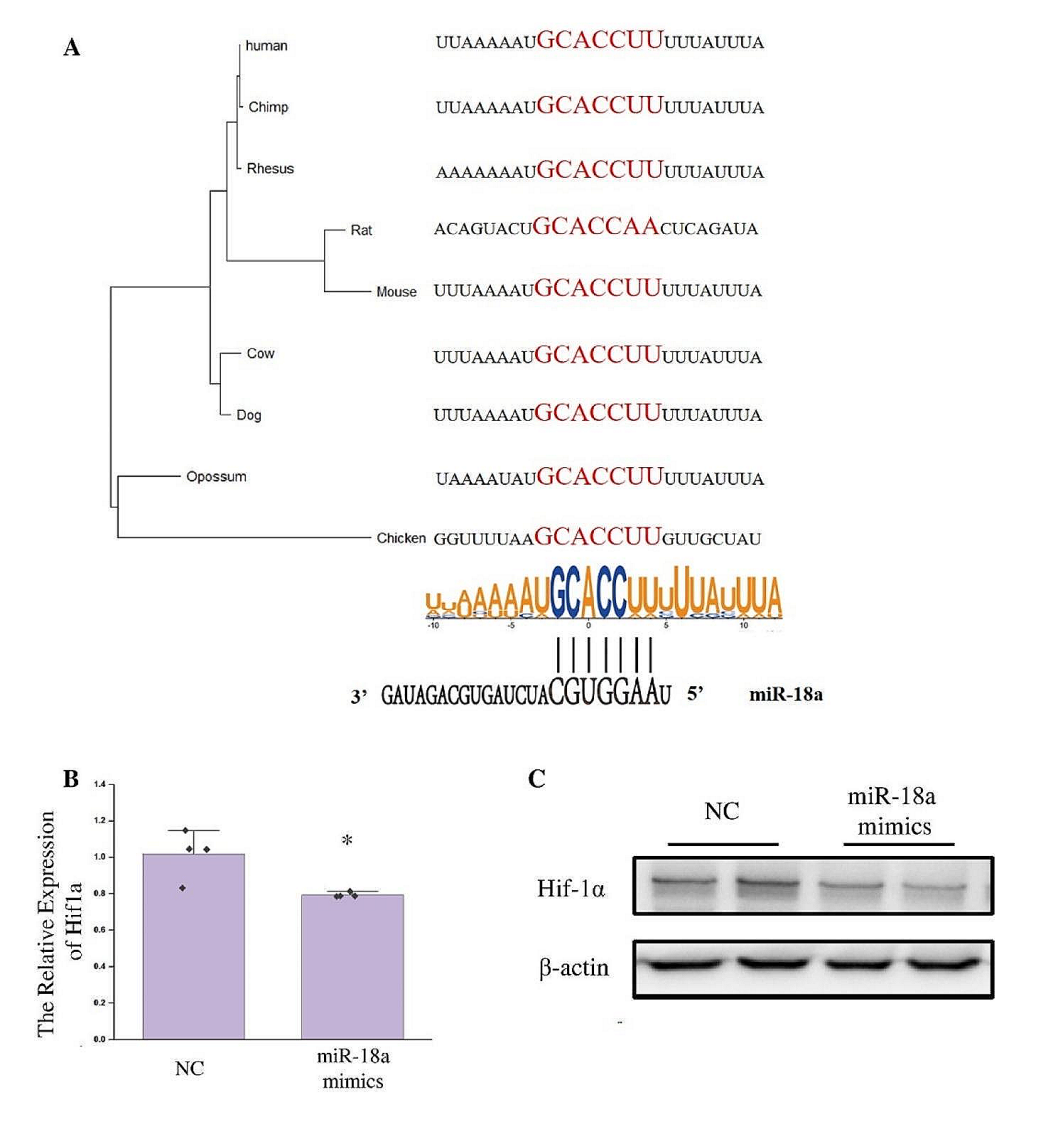
MiR-18a inhibited the expression of the *Hif1a* gene. (**A**) Phylogenetic tree constructed from the 3’UTRs of the *Hif1a* gene in multiple species. Analysis of miR-18 binding sites and flanking sequences in the 3’ UTRs of the *Hif1a* gene in multiple species. (**B**) The expression of the *Hif1a* gene was detected by qPCR after transfection of miR-18a mimics and NC for 24 h and hypoxic stress for 12 h. The *Actb* gene was used as the internal reference gene. *n* = 3, **: *p* < 0.01. (**C**) Western blot was used to detect the expression of Hif-1α after transfection of miR-18a mimics and NC for 24 h and hypoxic stress for 12 h. β-actin was used as an internal reference protein

We examined the 3’UTR of the *Hif1a* gene to further support the regulatory connection between miR-18a and the *Hif1a* gene. Genes may be subject to multiple regulation, and miRNAs mainly affect genes through the 3’UTR. For a particular gene, the selection pressure during its evolution may even originate primarily from the 3’UTR. The sequence homology of the 3’UTRs of the *Hif1a* gene in different species was analyzed (Fig. 4A). The phylogenetic tree based on the 3’UTRs of the *Hif1a* gene in different species was basically consistent with morphological classification. The complementary regions between the 3’UTRs of the *Hif1a* gene and the seed sequence of miR-18a were analyzed and shown (Fig. 4A). The complementary region was conserved in many species.

Then we investigated whether miR-18 could affect the *Hif1a* gene. After miR-18a mimics were transfected into HT22 cells for 24 h, the cells were exposed to hypoxic stress. The expression of the *Hif1a* gene was detected after 12 h of hypoxic stress (Fig. 4B, C). The *Hif1a* gene was shown to be inhibited by miR-18a at mRNA and protein levels.

**Fig. 5:**
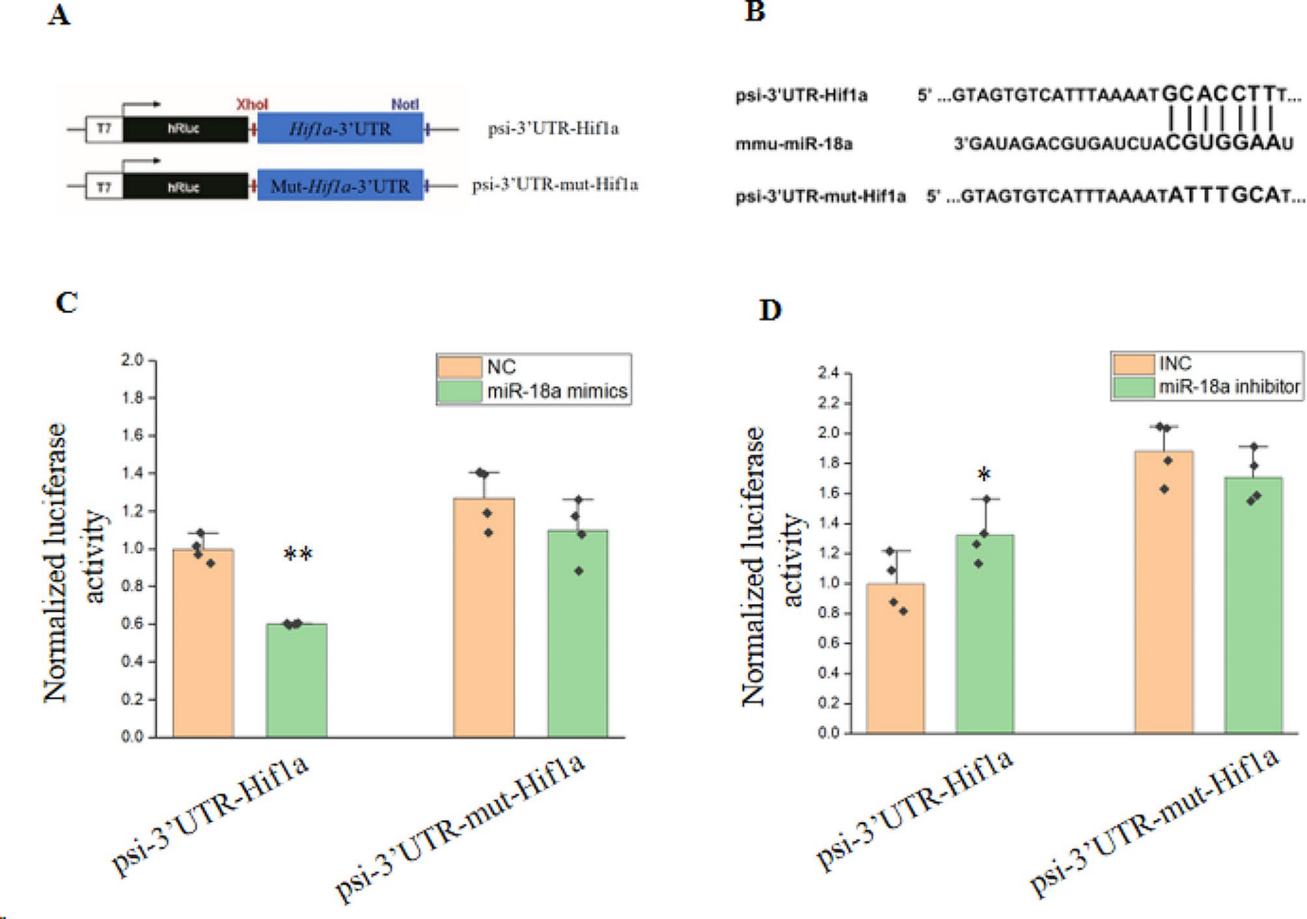
MiR-18a regulated the 3’UTR of the *Hif1a* gene. (**A**) Schematic of the psi-3’UTR-Hif1a vector and the psi-3’UTR-mut-Hif1a vector construction. (**B**) Schematic of the complementary sites between psi-3’UTR-Hif1a vector and psi-3’UTR-mut-Hif1a vector with the miR-18a seed sequence. (**C**) 24 h after co-transfection of miR-18a mimics (or NC) and the corresponding vector, the relative activity luciferase was detected. *n* = 4, **: *p* < 0.01. (D) 24 h after co-transfection of miR-18a inhibitor (or inhibitor negative control, lNC) and the corresponding vector, the relative activity luciferase was detected. *n* = 4, *: *p* < 0.05

We next tested whether miR-18a could directly target the complementary sequence of the *Hif1a* gene. The 3’UTR fragment of the *Hif1a* gene containing the predicted miR-18a binding site and a fragment with the mutated binding site were individually cloned into the 3’UTR of Renilla luciferase in the psiCHECK^TM^-2 vector (Fig. 5A, B). Luciferase activity was detected 24 h after co-transfecting miR-18a mimics and the corresponding vectors into HEK293T cells. The relative luciferase activity of HEK293T cells transfected with miR-18a mimics and the psi-3’UTR-Hif1a vector was significantly lower than that of HEK293T cells transfected with NC and psi-3’UTR-Hif1a vector (Fig. 5C). Co-transfection of miR-18a mimics and psi-3’UTR-mut-Hif1a vector did not significantly affect the relative luciferase activity. Co-transfection of miR-18a inhibitor and corresponding vectors also demonstrated that miR-18a could directly target the 3’UTR of the *Hif1a* gene (Fig. 5D).

### Hif-1α was involved in the regulation of glucose metabolism

**Fig. 6:**
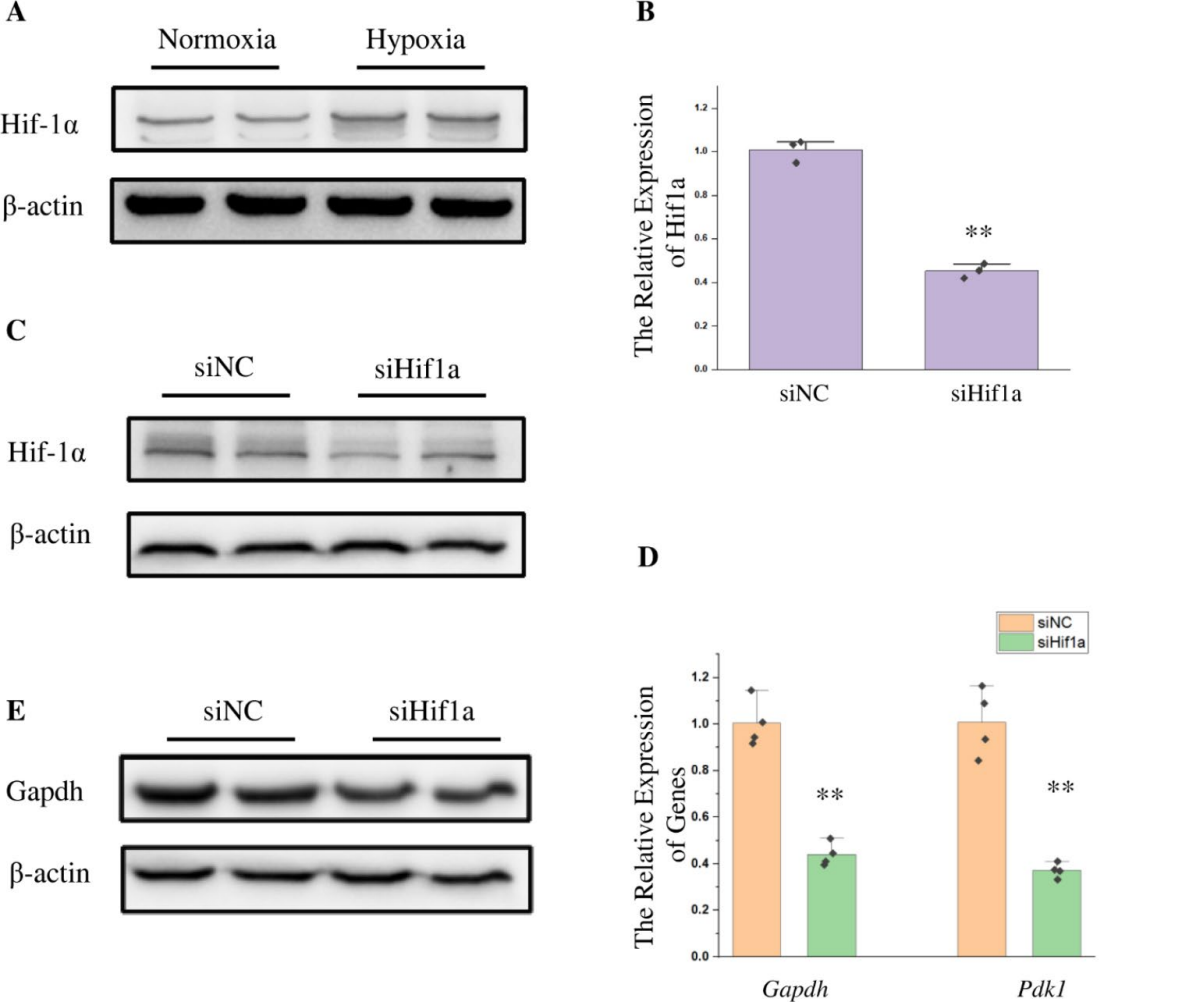
Hif-1α affected the expression of metabolism related genes. (**A**) Western blot was used to detect the expression of Hif-1α in HT22 cells after 12 h of hypoxic stress. (**B**) qPCR was used to detect the expression of the *Hif1a* gene after transfection of siRNA for 24 h and hypoxic stress for 12 h. The *Actb* gene was used as the internal reference gene. *n* = 3, **: *p* < 0.01. (**C**) Western blot was used to detect the expression of Hif-1α and Gapdh after transfection of siRNA for 24 h and hypoxic stress for 12 h. β-actin was used as an internal reference protein. (**D**) The *Gapdh* gene and *Pdk1* gene was detected by qPCR after transfection of siRNA for 24 h and hypoxic stress for 12 h. The *Actb* gene was used as the internal reference gene. *n* = 3, **: *p* < 0.01

Hif-1α is the most important hypoxia responsive factor, so miR-18a could affect glucose metabolism through the regulation of Hif-1α. Hif-1α has been shown to regulate glucose metabolism in response to hypoxic stress in several studies. Hypoxia increased the expression of Hif-1α in HT22 cells (Fig. 6A). The function of Hif-1α was verified in this study by siRNA. The inhibition efficiency was verified at the mRNA and protein levels (Fig. 6B, C). Then, 24 h after siRNA transfection, hypoxic stress was performed and the expression of related genes was measured. According to the qPCR results, cells transfected with *Hif1a* siRNA had lower relative expression levels of the *Gapdh* gene and *Pdk1* gene than cells transfected with negative control siRNA (Fig. 6D). The expression change of Gapdh in this process was detected at the protein level, and its expression was downregulated as Hif-1α expression was inhibited (Fig. 6C).

**Fig. 7:**
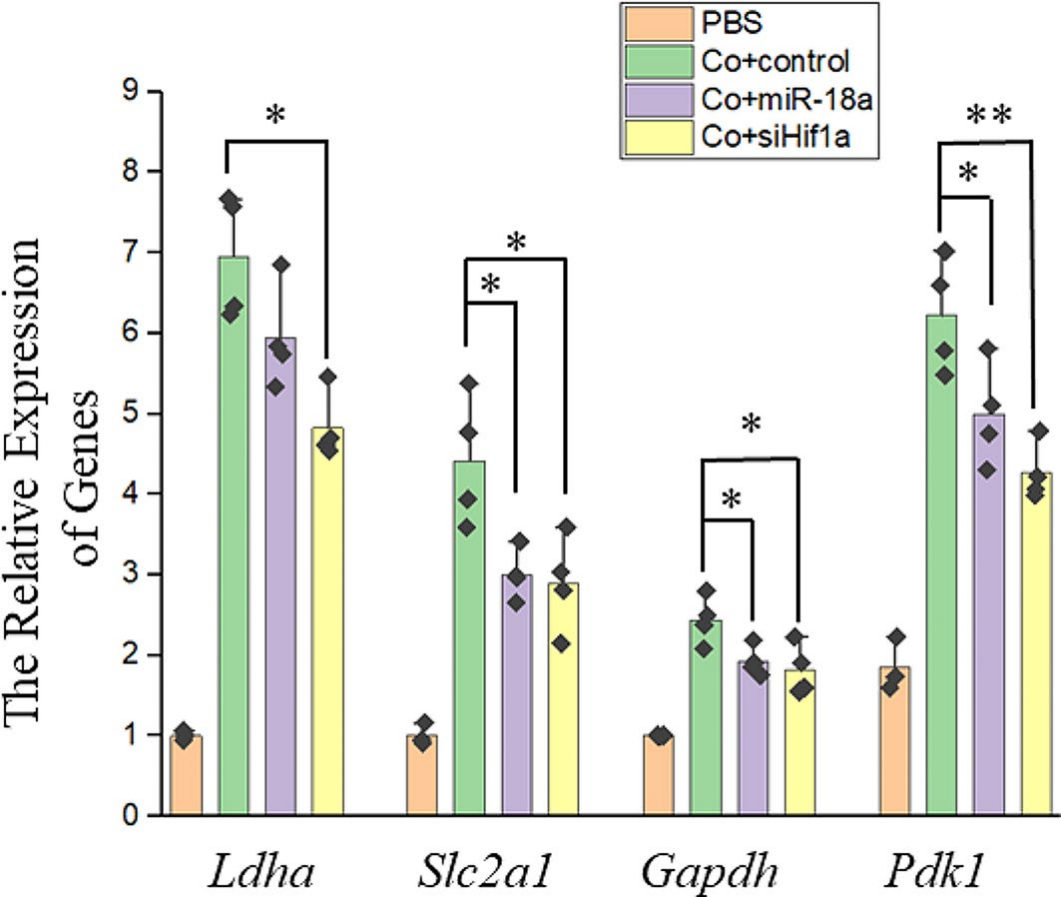
MiR-18a and Hif-1α were involved in the regulation of the glucose metabolism under CoCl_2_ stress. qPCR was used to assess the impact of miR-18a mimics and *Hif1a* siRNA on the expression of the *Ldha*, *Slc2a1*, *Gapdh* and *Pdk1* genes under CoCl_2_ stress. *n* = 4, *: *p* < 0.05, **: *p* < 0.01

CoCl_2_ treatment can mimic cell hypoxia. Therefore, we further verified the regulatory effect of miR-18a on glucose metabolism during CoCl_2_ stress. 6 h after transfection with miR-18a mimics or siRNA for *Hif1a*, the cells were treated with CoCl_2_. The expression of genes involved in the anaerobic glycolysis was examined (Fig. 7). CoCl_2_ stress caused an increase in the relative expression of the *Ldha*, *Slc2a1*, *Gapdh* and *Pdk1* genes. The transfection of miR-18a mimics or siRNA for *Hif1a* inhibited the corresponding genes. These findings suggested that miR-18a and Hif-1α are crucial regulators of the glucose metabolism under hypoxic stress.

## Discussion

We found that miR-18a could respond to hypoxic stress and was involved in regulating the expression of Hif-1α to affect glucose metabolism in neuronal cells. For the nervous system, hyperglycemia increases the risk of brain injury. Hyperglycemia is an important cause of cognitive deterioration, neurodegeneration and other neurological diseases [22]. Brain injury can also cause changes in blood glucose [23].Because of the key role of glucose regulation and glucose metabolism in physiological and pathological processes, especially in the process of hypoxia stress, we focused on the function of miR-18a in glucose metabolism in present work.

It has been shown that as a member of the miR-17-92 cluster, the occurrence and development of tumors are tightly correlated with miR-18a. MiR-18a is related to tumor metastasis in nasopharyngeal carcinoma [24], astroglioma [25], breast cancer [26] and other tumor cells. The role of miR-18a in the pathogenesis of disease remains a challenging topic. Among them, the results of miR-18a in glioma are controversial. It has been found that in glioma, miR-18a can inhibit cell proliferation by regulating RORA [27]. At the same time, some studies have proved that miR-18a promotes the occurrence and development of glioma by regulating the expression of CBX7 [28], HMBOX1 [29], and ALOXE3 [30]. Hypoxia, as a key factor in the tumor microenvironment, is also associated with the occurrence and development of tumors. Combined with the relationship between miR-18a, hypoxia and glioma, miR-18a may influence the occurrence and development of glioma by inhibiting the expression of HIF1A. As one of the most important hypoxia induced factors, HIF1A is closely related to the development of a variety of diseases, including glioma, and it can be used as a marker for the diagnosis and independent prognostic factor of disease [31]. For example, in diffuse gliomas, the up-regulated expression of HIF1A can affect T-cell exhaustion status [32]. In glioblastoma multiforme, MSH6 can affect the tumor microenvironment by regulating HIF1A to accelerate tumor metastasis [33]. Therefore, the function of miR-18a and HIF1A in disease development needs to be further investigated.

It’s crucial to find ways to decrease the damage that hypoxia causes to the central nervous system. Ischemic/hypoxic preconditioning is one of the important measures. HIF-1α and hypoxic signaling pathways have critical roles in Ischemic/hypoxic preconditioning [34]. Therefore, utilizing miR-18a mimics or inhibitor to regulate HIF-1α expression may have alleviating or therapeutic effects on neurological damage caused by hypoxia such as stroke. Therefore, miR-18a can be used as a marker for the diagnosis of stroke or cancer. MiR-18a can also be developed as a small molecule nucleic acid drug for the treatment of the above diseases. In order to better clinical application of miR-18a, the follow-up research will focus on the functional study of miR-18a in vivo.

MiRNAs are involved in the response to hypoxia in a variety of tissues, and the most important way of miRNA regulation is to affect metabolic transitions [35].Among the regulated metabolic pathways, the one that has received the most attention is glucose metabolism. Our manuscript also focused on miRNA and glucose metabolism. In the process of hypoxia research, the role of lipid metabolism has been gradually revealed [36]. Since Hif-1α can affect lipid metabolism, the role of miR-18a in the regulation of lipid metabolism during hypoxic stress needs to be investigated.

The *Gapdh* gene was often used as an internal reference gene when examining the regulatory effect of miR-18a on other genes. However, miR-18a can affect the expression of the *Gapdh* gene through Hif-1α. Therefore, the *Gapdh* gene is not suitable as an internal reference gene for functional studies of miR-18a. Similarly, using the *Gapdh* gene as an internal reference gene is not appropriate for other miRNAs that may influence Hif-1α, such as miR-135-5p, miR-17, etc.

In summary, we found that hypoxia caused down-regulation of miR-18a in HT22 cells. The change of miR-18a could regulate Hif-1α and affected glucose metabolism under hypoxic stress. MiRNAs are small molecules that naturally exist in the body and play a fine regulatory role in biological processes. So the functional study of miR-18a in hypoxia can provide a theoretical basis for the study and treatment of diseases such as stroke and other hypoxia induced neurodegenerative diseases.

## Data Availability

No datasets were generated or analysed during the current study.

## Abbreviations

HIF: Hypoxia-inducible factor
3’UTR: 3’untranslated region
VHL: Von Hippel–Lindau
EPO: Erythropoietin
VEGF: Vascular endothelial growth factor
DMEM: Dulbecco’s Modified Eagle Medium
PCR: Polymerase Chain Reaction
qPCR: Quantitative real-time Polymerase Chain Reaction
SEM: Standard error
NC: Negative control
PPI: Protein-protein interaction
